# Genomic signals found using RNA sequencing show signatures of selection and subtle population differentiation in walleye (*Sander vitreus*) in a large freshwater ecosystem

**DOI:** 10.1002/ece3.6418

**Published:** 2020-06-13

**Authors:** Matt J. Thorstensen, Jennifer D. Jeffrey, Jason R. Treberg, Douglas A. Watkinson, Eva C. Enders, Ken M. Jeffries

**Affiliations:** ^1^ Department of Biological Sciences University of Manitoba Winnipeg MB Canada; ^2^ Freshwater Institute, Fisheries and Oceans Canada Winnipeg MB Canada

**Keywords:** adaptive variation, gene flow, outlier loci, population genomics, transcriptomics

## Abstract

RNA sequencing is an effective approach for studying aquatic species yielding both physiological and genomic data. However, its population genetic applications are not well‐characterized. We investigate this possible role for RNA sequencing for population genomics in Lake Winnipeg, Manitoba, Canada, walleye (*Sander vitreus*). Lake Winnipeg walleye represent the largest component of the second‐largest freshwater fishery in Canada. In the present study, large female walleye were sampled via nonlethal gill biopsy over two years at three spawning sites representing a latitudinal gradient in the lake. Genetic variation from sequenced mRNA was analyzed for neutral and adaptive markers to investigate population structure and possible adaptive variation. We find low population divergence (*F*_ST_ = 0.0095), possible northward gene flow, and outlier loci that vary latitudinally in transcripts associated with cell membrane proteins and cytoskeletal function. These results indicate that Lake Winnipeg walleye may be effectively managed as a single demographically connected metapopulation with contributing subpopulations and suggest genomic differences possibly underlying observed phenotypic differences. Despite its high cost relative to other genotyping methods, RNA sequencing data can yield physiological in addition to genetic information discussed here. We therefore argue that it is useful for addressing diverse molecular questions in the conservation of freshwater species.

## Graphical abstract

Principal components analysis implemented in pcadapt, with color showing site and point shapes showing year collected. Out of 222,634 single nucleotide polymorphisms (SNPs) called from RNA seq data, 386 showed signatures of selection across a latitudinal gradient in Lake Winnipeg, Manitoba, Canada.

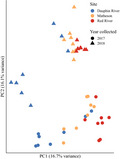

## 1 INTRODUCTION

Population abundances in aquatic systems are in decline globally, with a 36% decline in the marine Living Planet Index (LPI, http://livingplanetindex.org) and an 81% decline in the freshwater LPI between 1970 and 2012 (WWF, 2016). The LPI is an average rate of change in population size aggregated to the species level, based on population time‐series data. The decline in the freshwater LPI is especially alarming for those ecosystems, which cover 2.3% of the earth's global land surface area but are disproportionately high in species richness—for instance, one‐third of all described vertebrate species live in freshwater (Reid et al., 2019; WWF, 2018). It is therefore a significant concern that freshwater species are declining in abundance more rapidly than terrestrial or marine species (Reid et al., 2019). This decline underscores an urgent need for research supporting conservation efforts for these diverse freshwater organisms.

To take effective action, conservation practitioners require research on the environmental stressors a population faces, as well as population structure and evolutionary patterns to determine a population's adaptive potential (Connon, Jeffries, Komoroske, Todgham, & Fangue, 2018; Russello, Kirk, Frazer, & Askey, 2011; Waples & Gaggiotti, 2006). For instance, RNA sequencing and differential gene expression analysis can be used to conduct population risk assessments by identifying physiological thresholds, thus possibly informing management decisions (Connon et al., 2018). An advantage of using RNA sequencing for conservation research is that it provides information about both genetics and molecular physiology by returning transcript abundances and single nucleotide polymorphisms (SNPs) allowing researchers to gather a diverse array of information within one dataset. Moreover, a de novo transcriptome can be generated from a dataset of RNA reads using computational tools (e.g., Trinity described in Grabherr et al., 2011); therefore, researchers can take a set of samples collected in the field and develop a profile of physiological status and genomic patterns without relying on species‐specific probes or primers. These advantages make transcriptomics approaches useful for studying species of conservation concern, especially for species that do not have extensive molecular databases like those available for model species (e.g., zebrafish, *Danio rerio*).

Applications of RNA sequencing to address population genomics questions in nonmodel species are relatively less characterized compared to DNA sequencing. Several studies that have used RNA sequencing for population genomics describe domestication selection, such as in fish or plants (Christie, Marine, Fox, French, & Blouin, 2016; Gros‐Balthazard et al., 2019), while others address adaptive variation (Barts et al., 2018; Brown et al., 2018; Passow et al., 2017; Pratlong et al., 2015). Adaptive variation may be useful for conservation by delineating units with functional differences (Funk, McKay, Hohenlohe, & Allendorf, 2012; Russello et al., 2011). While many genomic methods rely on genes in linkage with outlier SNPs of interest to infer the functional significance of the SNP, the functional significance of SNPs in mRNA is often more readily interpretable because SNPs are more likely to occur in protein‐coding sequences than SNPs called from whole‐genome data (Verta & Jones, 2019). Within a transcript, the effects of SNPs in open reading frames can be predicted, which allows inferences of how protein function may be modified by genetic variation (Cingolani et al., 2012). Transcriptomics is then valuable for its potential to investigate the dynamic between phenotypic plasticity and evolution (Barts et al., 2018; DeBiasse & Kelly, 2016; Kelly, 2019; Passow et al., 2017; Pratlong et al., 2015).

A large topic of interest in conservation genomics is population structure or genomic divergence between different groups of individuals, which can support decisions on whether those groups should be managed as a single unit or several units (Funk et al., 2012). Here, population structure in RNA sequencing studies is often considered in the context of adaptation or functional variation (De Wit & Palumbi, 2013; Hoey & Pinsky, 2018), but relatively few transcriptomic studies make population structure a focus of their analyses. Therefore, RNA sequencing may be an effective method for characterizing physiological patterns, population structure, and adaptive variation in species and systems with little prior information available.

**Figure 1:**
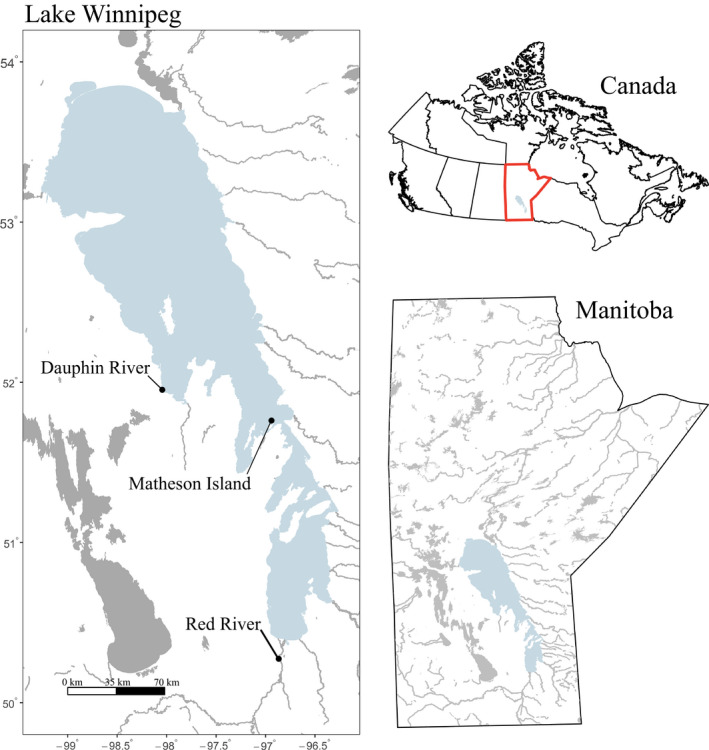
Sampling locations within Lake Winnipeg, Manitoba, Canada. Eight walleye (*Sander vitreus)* per year and per spawning site were collected, for *n* = 48 fish over 2017 and 2018. The Red River represents the south basin, Matheson Island represents the channel connecting the two lake basins, and the Dauphin River represents the north basin

Walleye (*Sander vitreus*) in Lake Winnipeg, Manitoba, are the largest component of the second‐largest freshwater fishery in Canada. Lake Winnipeg is characterized by a north and a south basin connected by a narrow channel (Johnston, Lysack, & Leggett, 2012; Figure 1). While previous microsatellite research showed limited population differentiation between groups in each basin except at two out of thirteen sampling locations (Backhouse‐James & Docker, 2012), morphological, life history, dietary, and environmental differences among Lake Winnipeg walleye suggest diverging genetic histories (Environment Canada, 2011; Johnson et al. 2012; Moles et al., 2010; Sheppard, Davoren, & Hann, 2015; Sheppard, Hann, & Davoren, 2018; Watkinson & Gillis, 2005). Within Lake Winnipeg, walleye have shown declining biomass and body condition, decreased catches, and commercial harvests above maximum sustainable yields for several years (Manitoba Government, 2018; Manitoba Sustainable Development, 2018). Observations of dwarf walleye suggest signs of selection against large, economically desirable fish, especially in the south basin of the lake (Johnston et al., 2012; Moles et al., 2010). One possibility is that phenotypic plasticity may underlie walleye responses to environmental differences between basins (Kelly, 2019), and this plasticity may be expressed as changes in walleye morphology, life history, and diet over time and across latitudes. An important first step in investigating possibly plastic patterns is to establish patterns of population structure underlying potential divergence, especially because genomic techniques can have higher resolution than microsatellite markers (Funk et al., 2012). In addition, plastic and evolutionary responses have overlapped in genes showing either changed expression or signatures of selection (Kelly, 2019). Therefore, information on population structure and biological differences in Lake Winnipeg walleye may help distinguish between plastic and evolutionary phenotypic responses, which may support future conservation efforts.

The current study aimed to show how mRNA sequencing can be an effective approach for developing critical pieces of information applicable to fisheries and conservation practitioners. We used RNA sequencing for genetic characterization of Lake Winnipeg walleye sampled from known spawning locations that potentially represent fish from the north and south basins. We also sampled fish collected at the channel that connects the north and south basins as an intermediate site. We hypothesized that walleye populations within Lake Winnipeg show evidence of distinct population differentiation identified using RNA sequencing data, despite the weak signatures from microsatellite data (Backhouse‐James & Docker, 2012). We predicted that the walleye population divergence may partially reflect the different environments and natural histories between the north and south basins of Lake Winnipeg.

## 2 METHODS

### 2.1 RNA extraction and sequencing

Gill tissue was collected from large (≥1.2 kg) predominately female (44 female, 4 unidentified sex) (Table S1) walleye over two years from three sites in the Lake Winnipeg system (Red River, Matheson Island, and Dauphin River, representing sites in the south basin, channel, and north basin, respectively; Figure 1; Table S1; *n* = 8 per year and site, *n* = 48 total). With *n* = 16 individuals per site over both years, sample sizes can predict mean population allele frequency by site, because sample sizes as low as *n* = 12 have been shown to be sufficient with RNA sequencing data (Schunter, Garza, Macpherson, & Pascual, 2014). These fish were sampled during the spawning season (approximately May through early June in 2017 and 2018). Walleye were collected by electrofishing, held in a live well for no longer than one hour, and anaesthetized using a Portable Electroanesthesia System (PES^™^, Smith Root) in accordance with approved animal use protocols of Fisheries and Oceans Canada (FWI‐ACC‐2017‐001, FWI‐ACC‐2018‐001), the University of Manitoba (F2018‐019), and the University of Nebraska‐Lincoln (Project ID: 1208). Fish were sampled nonlethally for gill tissue, where 2–3 mm of the terminal ends of 3–4 filaments from the left side each fish were collected and placed in RNA*later* (Thermo Fisher Scientific) that was kept at 4°C for 24 hr prior to storage at −80°C. Total RNA extractions were performed on gill tissue using RNeasy Plus Mini Prep Kits (QIAGEN) following manufacturer's protocols, with minor modifications (provided in the supplementary materials).

The quantity and quality of RNA were assessed with a Nanodrop One Spectrophotometer (Thermo Fisher) and electrophoresis on a 1% agarose gel, respectively. Total RNA was normalized to 80 ng/µl and sent to the McGill University and Génome Québec Innovation Centre sequencing facility (http://gqinnovationcenter.com) for cDNA library preparation and sequencing. A mRNA isolation approach was performed on 250 nanograms of total RNA using the NEBNext Poly(A) Magnetic Isolation Module (New England BioLabs). From mRNA, stranded cDNA libraries were created using the NEBNext Ultra II Directional RNA Library Prep Kit for Illumina (New England Biolabs). Each library was individually barcoded with NEBNext dual adaptors (New England Biolabs) prior to sequencing 100 base pair paired‐end reads. All 48 fish were sequenced on a single lane of a NovaSeq 6,000 (Illumina). 2.17 billion reads total were sequenced, with an average of 45,225,548 reads per sample collected (5,071,090 *SD*) (Table S1).

### 2.2 SNP calling

Raw read files were uploaded to the Graham and Cedar clusters on the Westgrid section of the Compute Canada partnership (https://www.westgrid.ca/). Read files were checked for quality using FastQC version 0.11.8 (Andrews, 2010) and trimmed using Trimmomatic version 0.36 (Bolger, Lohse, & Usadel, 2014). When using FastQC version 0.11.8 (Andrews, 2010), the program was set to allow two seed mismatches, a palindrome clip threshold of 30 nucleotides, and simple clip threshold of ten nucleotides. A sliding window size of 4 base pairs was used to filter data for a minimum Phred 64 quality of five, with five nucleotides trimmed from both the leading and trailing ends of reads, and a minimum read length of 36. After trimming, FastQC was used again to verify data quality. Scripts used for the analyses in this manuscript are provided at https://github.com/BioMatt/Walleye_RNAseq.

The SuperTranscripts pipeline was used to align reads (Davidson, Hawkins, & Oshlack, 2017) for SNP calling. First, Salmon version 0.11.3 (Patro, Duggal, Love, Irizarry, & Kingsford, 2017) was used to quantify read counts, as compared to a previously assembled reference transcriptome for walleye (Sequence Read Archive Accession SRP150633; Jeffrey et al., 2020). In Salmon, validate mappings, range factorization bins of size 4, sequencing bias, and GC bias options were all used, along with dumping equivalence classes for subsequent steps. Using the count estimates from Salmon, Corset version 1.07 (Davidson & Oshlack, 2014) was used to cluster the data for assembly into SuperTranscripts. A linear representation of the transcriptome was constructed with Lace version 1.00 (https://github.com/Oshlack/Lace) using information from Corset and the original transcriptome, where 263,272 unique gene features were identified using Trinotate (Grabherr et al., 2011) in the original transcriptome were gathered into 148,165 super clusters. Following Lace, STAR version 2.7.0a (Dobin et al., 2013) was used in 2‐pass mode to align trimmed reads to the reassembled transcriptome. Here, annotated junctions from Lace were provided along with the new transcriptome, and sjdbOverhang of 99 was chosen following recommended settings of 1 base pair below read length. A minimum of 79.6% reads uniquely mapped to the Lace‐clustered transcriptome (mean 81.5% ± 0.5% *SD*) (Table S1).

For calling SNPs, the STAR‐aligned reads were processed with Picard version 2.18.9 by adding read groups, splitting cigar ends, and merging bam files (Broad Institute, 2019); then, SNPs were called using FreeBayes version 1.2.0 (Garrison & Marth, 2012). Detailed methods for calling SNPs are provided in the Supplementary Materials. This resulted in 2,458,947 SNPs and 586,556 indels, which were used as unfiltered data for subsequent steps. We next filtered the VCF file from FreeBayes in two ways to (a) identify putatively neutral SNPs for population structure analyses and (b) SNPs for which neutrality was not assumed, using for outlier tests and functional analyses. Because purifying selection may be widespread throughout expressed RNA and we wished to study SNPs as close to neutrality as possible, several filtering steps were taken to create a putatively neutral SNP dataset (Gossmann et al., 2010). VCFtools version 0.1.14 was used to filter for biallelic SNPs of genotype and site quality ≥30, minimum minor allele frequency ≥0.05, no missing data in any individual, and in Hardy–Weinberg equilibrium with a *p*‐value < .005 (Danecek et al., 2011). SNPRelate version 1.16.0 was then used to prune SNPs for linkage disequilibrium at a threshold of 0.20, where super clusters were coded as chromosomes for the purposes of linkage disequilibrium pruning (Zheng et al., 2012). These steps resulted in a putatively neutral dataset of 52,372 SNPs used for population structure analyses. For a broader subset of SNPs for which neutrality was not assumed, VCFtools was used to filter for genotype and site quality ≥30, minimum minor allele frequency ≥0.05, and a maximum of two out of 48 possible individuals missing data. 222,634 SNPs were retained from these filtering steps, which were then used for outlier tests and functional analyses.

### 2.3 Population structure

To investigate population structure using the 52,372 putatively neutral SNPs, we used a combination of exploratory analyses, either with no prior information or with sampling location provided as priors, and population reassignment and differentiation tests to find genetic clusters despite possible signals of admixture or gene flow. Structure version 2.3.4 (Falush, Stephens, & Pritchard, 2003, 2007; Hubisz, Falush, Stephens, & Pritchard, 2009; Pritchard, Stephens, & Donnelly, 2000) was run with no prior location or population information, an initial value of alpha of 1.0, a maximum value of alpha of 10.0, prior mean *F*_ST_ of 0.01, lambda of 1.0, a burn in period of 10,000 repetitions, and 110,000 Markov Chain Monte Carlo repetitions after burn in. Structure plots were visualized with pophelper version 2.2.7 (http://royfrancis.github.io/pophelper/). Ten replicates of *K* = 2–5 were tested.

For analyses performed in R (R Core Team, 2019), the package vcfR was used to format genotype data for use with other programs (Knaus & Grünwald, 2017). Adegenet version 2.1.1 (Jombart, Devillard, & Balloux, 2010) was used in two ways: first, in an exploratory capacity to perform discriminant analysis of principal components (DAPC), where sampling location was provided for the DAPC as prior population information; second, population structure was investigated irrespective of sampling location by using cluster identification from successive K‐means, as implemented in the find.clusters function in Adegenet. Here, different numbers of clusters were explored in the data (40 principal components were retained for exploratory steps) and evaluated with a Bayesian information criterion (BIC), where the most well‐supported number of clusters with lowest BIC was 2 (Figure S1). In addition to exploring the two clusters, the population assignments from three clusters were used to explore genetic differentiation in the data because fish were sampled from three sites (Table S1). With Hierfstat version 0.04–22 (Weir & Cockerham, 1984; Yang, 1998), the Weir and Cockerham's pairwise *F*_ST_ was calculated among the three sampling locations, then between the two reassigned clusters described by Adegenet (Table S1). We generated 95% confidence intervals for these *F*_ST_ values in Hierfstat using a bootstrap approach over 1,000 iterations.

To visualize population differentiation, we used a PCA as implemented in Adegenet version 2.1.1 (Jombart et al., 2010) and t‐SNE as implemented in Rtsne version 0.15 (van der Maaten & Hinton, 2008). For the t‐SNE, two final dimensions were used, with 100 initial dimensions, 15 perplexity, theta of 0.5, and 5,000 iterations. Perplexity is, approximately, a measure of effective neighbors for a point (van der Maaten & Hinton, 2008). These approaches were used with the same settings applied to the putatively neutral SNPs, and visualizations were thus comparable between datasets.

### 2.4 Temporal stability & kinship

To test for temporal stability in the data, we created subsets of individuals caught in 2017 and 2018. As with the whole dataset, Weir and Cockerham's pairwise *F*_ST_ was calculated both among sampling locations and between the two reassigned clusters and generated 95% confidence intervals over 1,000 bootstrapped iterations in hierfstat (Table S1). Modest results that are consistent over time support confidence in a real genetic signal, as opposed to results driven by bias which are more likely to be inconsistent over time (Waples, 1998).

To address the possibility that sample collection, extraction, sequencing, or another process introduced an erroneous year effect into the data, we identified SNPs that differed between fish sampled in 2017 and 2018 with an *F*_ST_ above 0.01 using hierfstat and then filtered out those SNPs from the data using VCFtools version 0.1.14. The cutoff of 0.01 was chosen by only retaining those SNPs below an inflection point in the distribution of between‐year *F*_ST_ values. Following these steps, 13,640 SNPs (26.04% of 52,372 neutral SNPs total) were identified as having a large effect between years and were thus removed, leaving 38,732 SNPs. Analyses for population structure were then rerun with this smaller set of SNPs. *F*_ST_ was calculated both between sites and between two reassigned clusters described by Adegenet (Table S1). Data were also visualized by using a PCA as implemented in Adegenet version 2.1.1 and t‐SNE as implemented in Rtsne version 0.15.

To test whether our estimates of population structure were not driven by family groups (Waples, 1998), we used Colony version 2.0.6.4 to reconstruct pedigrees in our sample of 48 individuals, with consideration of possible full‐siblings (Jones & Wang, 2010; Wang, 2004). The putatively neutral SNPs were converted to the Colony format using a script by D. deWaters (https://github.com/dandewaters/VCF‐File‐Converter). The Colony command‐line input file was then generated to run the program with updated allele frequencies, dioecy, inbreeding possible, polygamy allowed, no clones, full sibship scaling, no sibship prior, unknown population allele frequencies, ten runs of medium length, full likelihood inference, and high precision. This Colony input file was generated using a script originally written by M. Ackerman (used in Ackerman et al., 2017), modified and posted with permission for the present study. Independent of Colony's maximum likelihood‐based approach, we also used the method of moments as implemented in SNPRelate version 1.18.0 (Zheng et al., 2012) to estimate a kinship coefficient between individuals, also using the putatively neutral SNPs.

### 2.5 Outlier SNPs

Using the full list of SNPs filtered for genotype quality 30, minor allele frequency >0.05 and two missing individuals allowed, but not filtered for Hardy–Weinberg equilibrium or linkage disequilibrium, we tested for outlier SNPs using an unsupervised approach in pcadapt version 4.1.0 (Luu, Bazin, & Blum, 2017). The unsupervised approach was used because weak population differentiation and the likely presence of admixed individuals in the data would either lower our sample size by filtering admixed individuals out or lead to false‐positive outlier loci by their inclusion when using a supervised approach with population structure included (Liu et al., 2017). While this may lead to issues of false positives from multiple tests (Foll & Gaggiotti, 2008), we addressed this issue by using a *q*‐value of 0.05 and focusing our interpretation on transcripts that contain two or more outlier SNPs. Two PCs were chosen for this analysis by observing the scree plot visualizing *K* = 1–20 following Cattell's rule, where the point that a smooth decrease in eigenvalues levels off on a scree plot is the last important PC for explaining the data (Cattell, 1966).

By relating the transcript ID of a significant outlier SNP (*q*‐value < 0.05) to that transcript's putative function and gene ID from the annotated reference transcriptome, a database of transcripts which diverged by sampling location or year was created for the Lake Winnipeg walleye in the present study. From this database, a list of transcripts relevant to either sampling location or year was used for gene set enrichment analysis using EnrichR (Chen et al., 2013; Kuleshov et al., 2016), thereby summarizing genes by gene ontology (GO) terms. In addition, transcripts were filtered to find those with two or more significant outlier SNPs that diverged by either sampling location or year, and these transcripts were few enough that enrichment analysis was not necessary. By only including genes with multiple outlier SNPs, we sought to reduce the presence of false‐positive signals in this outlier test.

## 3 RESULTS

### 3.1 Population structure

**Figure 2:**
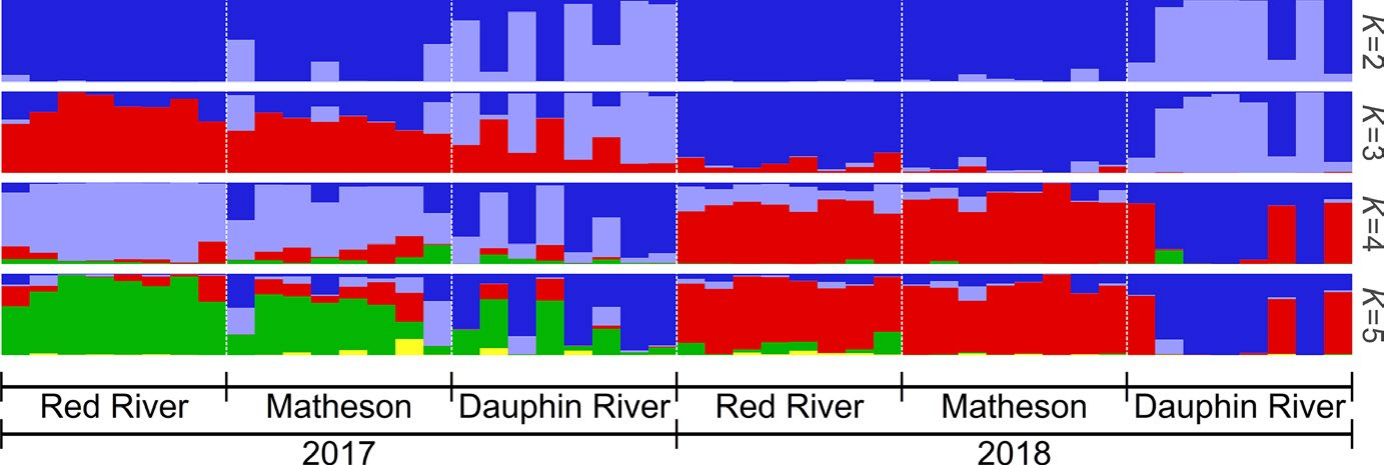
Representative Structure runs from ten replicates testing *K* = 2–5, organized by collection site (Red River in the south basin, Matheson Island in the channel, and Dauphin River in the north basin) and year collected (2017 and 2018) for all walleye (*Sander vitreus*) used in the present study. Collection site locations are available in Figure 1. This analysis was performed with 52,372 Hardy–Weinberg equilibrium filtered and linkage disequilibrium pruned, putatively neutral single nucleotide polymorphisms

**Figure 3:**
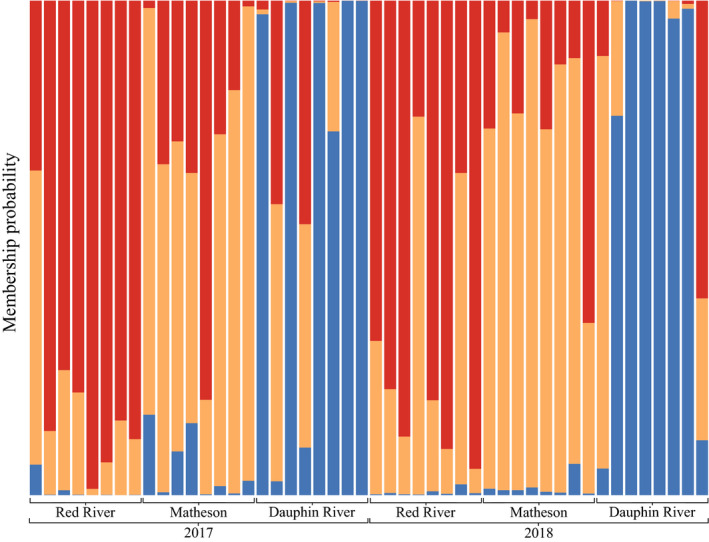
Membership probability plot of discriminant analysis of principal components using prior collection site information (Red River in the south basin, Matheson Island in the channel, and Dauphin River in the north basin) on walleye (*Sander vitreus*) collected over 2017 and 2018, performed using Adegenet. Collection site locations are available in Figure 1. This analysis was performed with 52,372 Hardy–Weinberg equilibrium filtered and linkage disequilibrium pruned, putatively neutral single nucleotide polymorphisms

Our data suggested weak but significant population structure between the north and south basins of Lake Winnipeg. The Red River and Matheson Island locations slightly diverged (*F*_ST_ = 0.0012), while the Dauphin River fish were the most genetically distinct group sampled (*F*_ST_ = 0.0068 and 0.0043 compared to the Red River and Matheson Island, respectively) (Figures 2, 3, Table 1, and Figure S2). Moreover, structure and the DAPC returned similar results with respect to which fish were admixed, although membership probabilities differed (Figures 2 and 3). Between *K* = 2–5, structure consistently separated the Dauphin River fish from the Matheson Island and Red River fish, while the Red River and Matheson Island fish did not separate from each other by site, but instead separated between years (Figure 2).

**Table 1:** Weir and Cockerham's pairwise *F*_ST_ calculated with hierfstat between the Red River in the south basin, Matheson Island in the channel, and Dauphin River in the north basin for all 48 walleye (*Sander vitreus*) sampled in both 2017 and 2018. 95% confidence intervals are provided in parentheses

	Red River (south basin)	Matheson Island (channel)	Dauphin River (north basin)
Red River	–	0.0012 (0.0009–0.0016)	0.0068 (0.0064–0.0074)
Matheson Island		–	0.0043 (0.0039–0.0048)
Dauphin River			–

Note

Collection site locations are available in Figure 1. This analysis was performed with 52,372 Hardy–Weinberg equilibrium filtered and linkage disequilibrium‐pruned, putatively neutral single nucleotide polymorphisms.

The PCA and t‐SNE used with the putatively neutral SNPs show similar patterns of Matheson and Red River fish separated, but more similar to each other than either with the Dauphin River fish (Figure S2). When comparing the PCA and t‐SNE plots between the neutral linkage disequilibrium‐pruned SNPs and the broader collection of SNPs used for outlier analyses, genetic differentiation between the Red River and Matheson Island fish disappears when using all of the SNPs with the t‐SNE, whereas separation between the two sites persists when only using Hardy–Weinberg equilibrium filtered and LD‐pruned SNPs.

### 3.2 Population assignment

Using two clusters for reassignment from Adegenet, out of 48 fish, 36 clustered in one group (Cluster 1) and twelve in the other (Cluster 2; Table S1). Cluster 1 was characterized by a combined Red River and Matheson Island group of fish with few Dauphin River fish (six were collected from the Dauphin River, 14 from Matheson Island, and 16 from the Red River), while Cluster 2 was characterized by Dauphin River fish and a small number of Matheson Island fish (ten fish from the Dauphin River and two from Matheson Island). Weir and Cockerham's pairwise *F*_ST_ between these two reassigned clusters was 0.0095 with a 95% confidence interval between 0.0090–0.010.

Using three clusters for reassignments from Adegenet, out of 48 fish, 19 were in one group (Cluster 1), ten fish were in another group (Cluster 2), and 19 fish in a final group (Cluster 3). Clusters 1 and 3 were characterized by a year effect, where every individual in Cluster 1 was captured in 2018 and every individual in Cluster 3 was captured in 2017. Both Clusters 1 and 3 had 16 out of 19 fish coming from the Red River or Matheson Island sites. Meanwhile, all ten fish in Cluster 2 were from the Dauphin River with five fish each collected in 2017 and 2018 (Table S1).

### 3.3 Temporal stability and kinship

When partitioning individuals by sampling location and year collected, all confidence intervals for between‐site pairwise *F*_ST_ estimates overlapped over both sampling years, indicating consistent patterns of between‐site divergence in 2017 and 2018. However, values between the Dauphin River and Matheson Island varied the most, with an estimate of 0.0044 (0.0035–0.0052) in 2017, and 0.0060 (0.0051–0.0070) in 2018 (Table 2).

**Table 2:** Weir and Cockerham's Pairwise *F*_ST_ calculated with hierfstat between the Red River in the south basin, Matheson Island in the channel, and Dauphin River in the north basin. *F*_ST_ values above the diagonal represent the 24 walleye (*Sander vitreus*) collected in 2017, and values below the diagonal represent the 24 collected in 2018

	Red River (south basin)	Matheson Island (channel)	Dauphin River (north basin)
Red River	–	0.0023 (0.0015–0.0031)	0.0073 (0.0064–0.0082)
Matheson Island	0.0019 (0.0011–0.0028)	–	0.0044 (0.0035–0.0052)
Dauphin River	0.0067 (0.0058–0.0077)	0.0060 (0.0051–0.0070)	–

Note

95% confidence intervals are provided in parentheses. Collection site locations are available in Figure 1. This analysis was performed with 52,372 Hardy–Weinberg equilibrium filtered and linkage disequilibrium pruned, putatively neutral single nucleotide polymorphisms.

Using the 38,732 SNPs filtered for loci, which showed *F*_ST_ between years of >0.01, *F*_ST_ between the two reassigned clusters found using Adegenet (Table S1) was 0.010 (0.0094–0.011). With these same year effect‐filtered SNPs, pairwise *F*_ST_ between sites did not significantly differ from values found using the neutral SNPs either overall or in a subset by year (Table S2). The PCA and t‐SNE on the SNPs filtered for a year effect showed patterns of spatial differentiation consistent with other analyses, with the Dauphin River fish being more separate from the Red River and Matheson Island group of fish (Figure S3).

We found no evidence of kinship using either Colony or the method of moments. Over ten replicate runs in Colony, individuals belonged to separate families with inclusive and exclusive probabilities of 1.0000 each. Using the method of moments implemented in SNPRelate (Zheng et al., 2012), the highest kinship coefficient between two individuals was 0.096 (mean 0.053 ± 0.019 *SD*), where a kinship coefficient of approximately 0.5 would indicate full‐siblings.

### 3.4 Outlier SNPS

**Figure 4:**
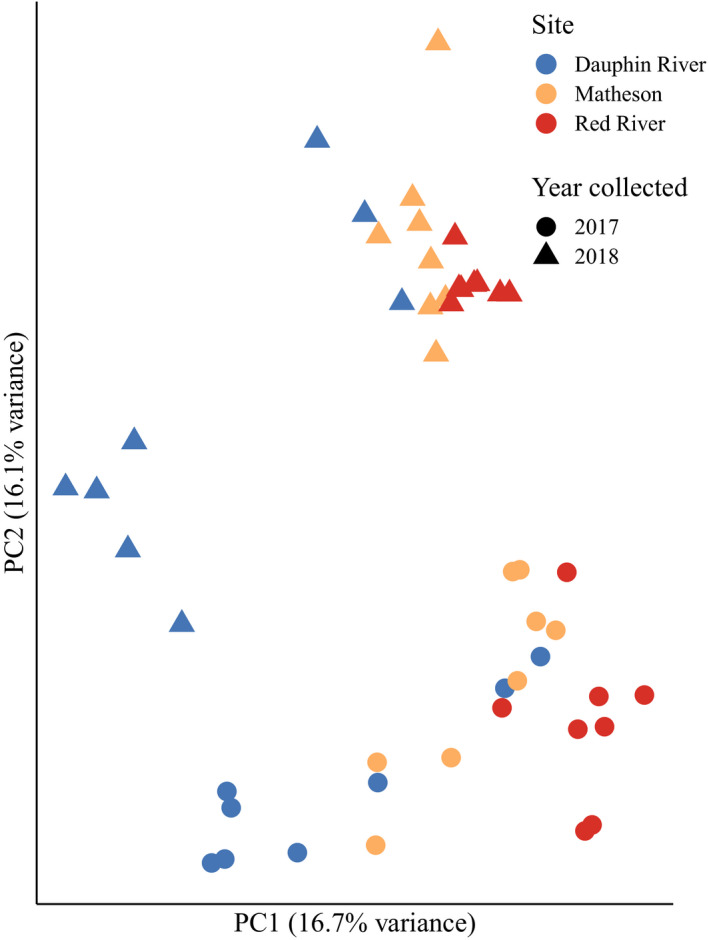
Principal components analysis implemented in pcadapt with color showing site collected (red for Red River in the south basin, yellow Matheson Island in the channel, and blue Dauphin River in the north basin), circles showing walleye (*Sander vitreus*) collected in 2017, and triangles showing walleye collected in 2018. Collection site locations are available in Figure 1. This analysis was performed using a set of 222,634 single nucleotide polymorphisms (SNPs) filtered for quality, minor allele frequency >0.05, and a maximum of two out of 48 missing individuals, but not filtered for Hardy–Weinberg equilibrium or pruned for linkage disequilibrium. These SNPs are, thus, more likely to represent patterns of adaptive variation in the system, and outlier analyses were performed using this set of SNPs. Principal component 1 (PC1) represents a latitudinal gradient, while principal component 2 (PC2) represents a genetic divergence between sampling years. Outlier SNPs that contribute to each of these axes were selected for functional analyses (Tables 3 and 4), with 386 SNPs contributing to PC1 and 791 SNPs contributing to PC2, significant at Benjamini–Hochberg adjusted *p*‐values < .05

There was site‐specific differentiation across principal component 1 (PC1) in the pcadapt analysis (Figure 4). In total, 1,177 SNPs were outliers at *q* < 0.05, with 386 SNPs contributing to PC1 where fish separated by site, and 791 SNPs contributing to PC2 where fish separated by year (Figure 4). For the 386 SNPs associated with PC1 (Figure 4), 120 uniquely annotated transcripts were available for enrichment analysis using EnrichR. These transcripts corresponded to GO terms such as purine ribonucleoside triphosphate binding, ATP‐binding, and adenyl ribonucleotide binding, all significant at Benjamini–Hochberg adjusted *p*‐values < .05 (Table S3). By filtering for uniquely annotated transcripts with ≥2 outlier SNPs associated with PC1, 19 transcripts were identified (Table 3) that varied by sampling location. Six of these genes were associated with ion channels and cell membrane transport, including claudin‐10, ankyrin‐3, sodium/hydrogen exchanger 6, sodium/potassium‐transporting ATPase subunit alpha‐3, perforin‐1, and ATP‐binding cassette subfamily A member 12. Additionally, four genes that varied spatially were associated with the cytoskeleton, such as myosin‐9, beta/gamma crystallin domain‐containing protein 1, tubulin beta‐4B chain, and interferon‐induced protein 44.

**Table 3:** Genes that vary along a latitudinal gradient in Lake Winnipeg walleye (*Sander vitreus*) with ≥ 2 outlier single nucleotide polymorphisms (SNPs) from pcadapt, each significant at a Benjamini*–*Hochberg adjusted *p*‐value < .05 (PC1 in Figure 4)

SwissProt gene name	Protein	Summary function
ABCA12	ATP‐binding cassette subfamily A member 12	Cell Membrane
ANK3	Ankyrin−3	Cell Membrane
atp1a3	Sodium/potassium‐transporting ATPase subunit alpha−3	Cell Membrane
CLDN10	Claudin−10	Cell Membrane
Prf1	Perforin−1	Cell Membrane
SLC9A6	Sodium/hydrogen exchanger 6	Cell Membrane
CRYBG1	Beta/gamma crystallin domain‐containing protein 1	Cytoskeleton
IFI44	Interferon‐induced protein 44	Cytoskeleton
MYH9	Myosin−9	Cytoskeleton
TUBB4B	Tubulin beta−4B chain	Cytoskeleton
DNASE1L1	Deoxyribonuclease−1‐like 1	DNase
EIF4G1	Eukaryotic translation initiation factor 4 gamma 1	Expression regulation
srebf2	Sterol regulatory element‐binding protein 2	Expression regulation
Znf879	Zinc finger protein 879	Expression regulation
CLPX	ATP‐dependent Clp protease ATP‐binding subunit clpX‐like, mitochondrial	Protease
CXCR3	C‐X‐C chemokine receptor type 3	Signaling
MATK	Megakaryocyte‐associated tyrosine‐protein kinase	Signaling
Ralgds	Ral guanine nucleotide dissociation stimulator	Signaling
Pol	LINE−1 retrotransposable element ORF2 protein	Transposable Element

Note

SwissProt gene names and corresponding proteins are provided, and general cellular location or function of these genes is described in Summary Function. This analysis was performed using a set of 222,634 single nucleotide polymorphisms (SNPs) not filtered for Hardy–Weinberg equilibrium or pruned for linkage disequilibrium, unlike the putatively neutral set of SNPs used for population structure analyses.

Using the 791 SNPs associated with principal component 2 (PC2), which varied by year (Figure 4), 130 uniquely annotated transcripts were available for enrichment analysis; however, no GO terms were significant at an adjusted *p*‐value < .05. For transcripts with ≥2 PC2 outlier SNPs, 17 uniquely annotated genes were identified of which six were either transposons, transposable elements, or fragments of transposons (Table 4). Two genes that code for the proteins serine/threonine‐protein phosphatase 6 catalytic subunit and protein BTG3, which regulate cell division in the G1 to S phase transition, were also identified (Table 4).

**Table 4:** Genes that vary between 2017 and 2018 in Lake Winnipeg walleye (*Sander vitreus*) with transcripts containing ≥2 outlier single nucleotide polymorphisms (SNPs) from pcadapt, each SNP significant at a Benjamini*–*Hochberg adjusted *p*‐value < .05 (PC2 in Figure 4)

SwissProt Gene Name	Protein	Summary Function
Gp1bb	Platelet glycoprotein Ib beta chain	Cell Adhesion
MFAP4	Microfibril‐associated glycoprotein 4	Cell Adhesion
BTG3	Protein BTG3	Cell Division
Ppp6c	Serine/threonine‐protein phosphatase 6 catalytic subunit	Cell Division
Slc12a3	Solute carrier family 12 member 3	Cell Membrane
MMD	Monocyte to macrophage differentiation factor	Expression regulation
Srsf10	Serine/arginine‐rich splicing factor 10	Expression regulation
Ube2a	Ubiquitin‐conjugating enzyme E2 A	Expression regulation
Znf18	Zinc finger protein 18	Expression regulation
St6galnac2	Alpha‐*N*‐acetylgalactosaminide alpha−2,6‐sialyltransferase 2	Protein Modification
MYO9A	Unconventional myosin‐IXa	Signaling/Cytoskeleton
Pol	LINE−1 retrotransposable element ORF2 protein	Transposable Element
RTase	Probable RNA‐directed DNA polymerase from transposon BS	Transposable Element
TC1A	Transposable element Tc1 transposase	Transposable Element
TN6	Putative transposase in *Dicentrarchus labrax* (European seabass)	Transposable Element
TY3B‐G	Transposon Ty3‐G Gag‐Pol polyprotein	Transposable Element
YTX2	Transposon TX1 uncharacterized protein (Fragment)	Transposable Element

Note

SwissProt gene names and corresponding proteins are provided, and general cellular location or function of these genes is described in Summary Function. This analysis was performed using a set of 222,634 single nucleotide polymorphisms (SNPs) not filtered for Hardy–Weinberg equilibrium or pruned for linkage disequilibrium, unlike the putatively neutral set of SNPs used for population structure analyses.

## 4 DISCUSSION

We observed weak population structure characterized by groups collected at the Red River and Matheson Island sampling locations, representing south basin and channel fish, contrasted with a group collected at the Dauphin River, representing north basin fish. As such, the north and south basin walleye in Lake Winnipeg may be separate groups with an *F*_ST_ of 0.0095, but with gene flow between them primarily at the channel connecting the two basins. Consistent with results in the present study, a study using microsatellites found a similar weak, but significant, differentiation between Lake Winnipeg walleye from some sites in the north and south basins (e.g., *F*_ST_ = 0.022 between the Grand Rapids in the north and Red River in the south; Backhouse‐James & Docker, 2012), suggesting genomic divergence between walleye from the two basins. However, microsatellite data did not resolve overall population structure between basins to a fine‐scale as in the present study. That is, microsatellites were limited to describing differentiation between particular sites, while population reassignment with SNPs from RNA data enabled descriptions of structure in the context of straying and possible metapopulation structure characterized by demographic connectivity. While facilitated gene flow has not been reported for Lake Winnipeg walleye (Manitoba Government, 2018; Manitoba Sustainable Development, 2018), historical stocking programs may have contributed to weak population structure within Lake Winnipeg by introducing walleye from nearby Lake Manitoba (Backhouse‐James & Docker, 2012). This unknown amount of gene flow from other systems, including up to 26.5 million fish annually between 1970 and 1983 (Lysack, 1986), may have masked signatures of spatial population differentiation in Lake Winnipeg. While an estimated *F*_ST_ of 0.0095 between separate groups is low, there is reason to believe that widespread purifying selection in RNA data may decrease *F*_ST_ estimates in comparison with estimates generated with microsatellites, consistent with observations of negative selection in protein‐coding sequences of many plant species (Gossmann et al., 2010).

### 4.1 Temporal differentiation

Differentiation between years was strongest in the south basin, where the Red River and Matheson Island fish separated by year to a greater extent than the Dauphin River walleye. Three hypotheses may explain these patterns of stronger temporal differentiation in the south basin. First, a cohort effect may underlie this pattern, where different year classes were more strongly represented in the lake during a given sampling year. A cohort effect could be the result of greater fishing pressure in the south basin than the north basin, as indicated by smaller allowed net mesh sizes in the south basin (Manitoba Sustainable Development, 2019). Fishing pressure can change population dynamics and age structure in exploited species (Anderson et al., 2008; Murphy & Crabtree, 2001); therefore, large fisheries operating since at least 1,890 may have affected age structure in Lake Winnipeg fish (Department of Fisheries, 1891). Cohort effects may alternatively be influenced by environmental conditions including predation intensity, water temperature, and time to hatch as observed in Lake Erie, Oneida Lake, and Lake Huron walleye (Busch, Scholl, & Hartman, 1975; Fielder, Schaeffer, & Thomas, 2007; Forney, 1976). A second hypothesis is that some Lake Winnipeg walleye may engage in unobserved skipped spawning or alternate year spawning, which have been unexpectedly found in several species and may be present in walleye (Carlander, Whitney, Speaker, & Madden, 1960; Henderson, Wong, & Nepszy, 1996; Moles, Johnston, Robinson, Leggett, & Casselman, 2008; Rideout & Tomkiewicz, 2011). Third, the observed year effect may be an artifact of error introduced during sampling, extraction, sequencing, or bioinformatics. While some error contributing to between‐year differentiation is impossible to rule out, the possibility that a particular analysis or filtering method introduced the year effect is reasonably small given that distinct tests showed consistent year effects. Moreover, the data reveal a consistency in spatial patterns with and without the year effect, demonstrating that at least the spatial population differentiation in Lake Winnipeg walleye is likely real.

While pairwise site *F*_ST_ values were temporally consistent (i.e., no significant differences between years), the greatest pairwise *F*_ST_ confidence interval difference between years was between walleye collected at Matheson Island and the Dauphin River, where confidence intervals for *F*_ST_ estimates overlapped by only 0.0001 between 2017 and 2018. Following Amrhein, Greenland, and McShane (2019), we interpret here the possibility that the entire range of these confidence intervals reflects meaningful patterns in the data and that *F*_ST_ between the Dauphin River and Matheson Island was different between 2017 and 2018. Because Matheson Island represents a narrow channel connecting the north and south basins of Lake Winnipeg, fish which would normally spawn in the Dauphin River may have used the channel more often in 2017, thus, lowering *F*_ST_ when performing a site‐wise comparison. This difference in habitat use may have arisen from an undetermined environmental variable, such as time of ice melt, which in the north basin was ten days later in 2018 than in 2017 (D. Watkinson, unpublished data found using https://zoom.earth/). Nevertheless, confidence intervals for *F*_ST_ overlapped between years between the Matheson Island and Dauphin River sites; therefore, patterns of environment‐dependent spawning site fidelity must be corroborated with other data, such as by telemetry.

Notably, gene flow appears to be one way from the southern Red River, northward. Going by capture location, no fish caught in the Red River showed a genetic background consistent with the Dauphin River fish, while with the Adegenet‐reassigned clusters, no fish assigned to the mostly Dauphin River group was found in the Red River. On the other hand, fish which showed a genetic background consistent with the Red River group were found in the Dauphin River, both based on capture location and population reassignment.

### 4.2 Biological significance

Several studies report morphological and life‐history differences between basins in Lake Winnipeg walleye consistent with the two delineated groups found in this study. Furthermore, environmental data show a north–south basin distinction with temperature, turbidity, mean depth, suspended solid, sulfate, sodium, chloride, and nutrient differences between the two basins (Brunskill, Elliot, & Campbell, 1980; Environment Canada, 2011). Walleye in the south basin show a bimodal growth pattern, where fisheries‐induced selection may have contributed to the observation of dwarf walleye (Johnston et al., 2012; Moles et al., 2010; Sheppard et al., 2018). Harvest‐induced genetic changes have been linked to size reductions in other walleye within two generations (Bowles, Marin, & Fraser, 2019). If walleye were panmictic throughout Lake Winnipeg, we might expect the dwarf morphotype to occur with similar frequency in the north basin. However, out of 616 total walleye caught in 2010 and 2011 (178 in the north basin, 438 in the south basin), only two out of 32 dwarf fish were caught in the north basin (Sheppard et al., 2018). Diet has also been shown to differ between north and south basin walleye, possibly because of prey or turbidity differences between the two basins (i.e., higher turbidity in the south basin) (Brunskill et al., 1980; Sheppard et al., 2015). Between 1979 and 2003, population characteristics such as age and length at 50% maturity were higher, while growth rate was slower in the north basin walleye, suggesting some level of isolation among walleye between basins (Johnston et al., 2012). These population characteristics may no longer be higher in the north basin following the collapse of the rainbow smelt (*Osmerus mordax)*, after which walleye body condition has decreased since 2010 (Manitoba Government, 2018). Scale morphometry further suggests differences among spawning aggregations of walleye, especially between the north and south basins (Kritzer & Sale, 2004; Watkinson & Gillis, 2005). Taken together, the results of our study and those of previous studies suggest weak population structure among Lake Winnipeg walleye, with differentiation between walleye in the north and south basins. This pattern of weak population structure, high connectivity, but biologically significant differentiation is common in marine fishes such as the Atlantic cod (*Gladus morhua*) or Atlantic salmon (*Salmo salar*) (Aykanat et al., 2015; Knutsen et al., 2011), and of other walleye such as those observed in Lake Erie (Chen et al., 2020; Stepien, Snyder, & Knight, 2018).

The results of the present study suggest that the genetic differences between Lake Winnipeg walleye populations may have functional consequences. Out of 19 transcripts that had multiple SNPs that varied by sampling location, eight were related to membrane function, particularly ion channel activity. One of these proteins, Claudin‐10 mRNA expression levels have been related ammonia exposure (Connon, Deanovic, Fritsch, D’Abronzo, & Werner, 2011), rearing density (Sveen et al., 2016), and salinity (Bossus, Madsen, & Tipsmark, 2015; Kolosov, Bui, Chasiotis, & Kelly, 2013; Marshall et al., 2018) in fishes. Spatial variation in cell membrane proteins is consistent with environmental differences between basins in chemicals such as sodium, chloride, and phosphorous (Environment Canada, 2011), although the biological impacts of these spatial chemical differences are unknown. Four cytoskeletal proteins were represented in the outlier SNPs that vary by sampling location as well. Cytoskeletal function is connected to cell growth in plants (Hussey, Ketelaar, & Deeks, 2006; Wasteneys & Galway, 2003), yeast (Li, Zheng, & Drubin, 1995; Pruyne & Bretscher, 2000), mouse cells (Kim & Coulombe, 2010; Kim, Wong, & Coulombe, 2006), and zebrafish (Johnston, Bower, & Macqueen, 2011). Spatial variation in genes related to cell growth may thus be consistent with growth rate differences observed among walleye in Lake Winnipeg, where north basin fish had higher growth rates in 2010 and 2011 (Sheppard et al., 2018). Though plastic phenotypes are likely important for walleye responses to environmental differences (Kelly, 2019), the outlier loci found in the present study provide some evidence for adaptive divergence despite weak population differentiation. While the biological significance of the outlier SNPs has not been confirmed, the SNPs themselves or the genes they reside in may be the focus of future research.

### 4.3 Limitations

Despite its advantages, there are some limitations to using mRNA sequencing in the context of population genetics. The depth of sequencing required for differential gene expression and differential exon usage leads to greater costs associated with mRNA sequencing studies relative to reduced representation methods such as RAD‐seq (Davey & Blaxter, 2010). This often translates to a lower sample size, as is the case in our study. Reduced sample sizes can bias aspects of population genetic analyses, including identifying population structure (Waples & Gaggiotti, 2006) and outlier SNPs (Luu et al., 2017), although sample sizes as low as 12 yield accurate mean population allele frequencies with mRNA sequencing data (Schunter et al., 2014). Second, mutations in mRNA are widely under selection (Chamary & Hurst, 2005); therefore, caution must be exercised when interpreting SNPs from mRNA in genetic tests assuming neutrality. Third, linkage disequilibrium is useful for analyses of selective sweeps and demographic history (Catchen et al., 2017; Garrigan & Hammer, 2006; Hoffmann & Willi, 2008), among other approaches, but mRNA data may not be appropriate for these analyses because the extent of linkage between transcripts and marker density is difficult to characterize for species without published chromosome‐level genome assemblies. Finally, one key element of how transcriptomics was used in the present study is that it measured expressed mRNA in gill tissue. Messenger RNA expression provides useful information for transcript quantification‐based analyses, but likely biases SNP discovery toward more highly expressed transcripts in the tissue collected. It is unknown how this expression‐specific bias may influence population genomics. Nevertheless, mRNA sequencing has proven useful for recapitulating population structure discovered with traditional genetic methods (Jeffries et al., 2019) and describing previously uncharacterized population structure (Ellison et al., 2011; Yan et al., 2017).

### 4.4 Conservation applications

We used population genetics and outlier detection to characterize weak, but biologically significant population structure, possible one‐way gene flow, and genetic variation possibly underlying biological differences among Lake Winnipeg walleye. These results are consistent with observations of behavioral differences leading to fine‐scale divergence in the walleye of other systems (Stepien, Murphy, Lohner, Sepulveda‐Villet, & Haponski, 2009). The low levels of population differentiation and possible gene flow from the south basin northward indicate that this system may be effectively managed as a demographically connected metapopulation with two contributing populations (Kritzer & Sale, 2004), consistent with conclusions from scale morphology presented in Watkinson and Gillis (2005) and with observations of subtle stock structure in Lake Erie walleye (Chen et al., 2020; Stepien et al., 2018).

The results from this study provide valuable information for walleye management, especially because the status of Lake Winnipeg walleye is becoming a concern and conservation action may be necessary to sustain the fishery. Signs of a declining fishery include a decrease in biomass and body condition between 2010 and 2015 (Manitoba Government, 2018), possible unnatural selection against larger, economically desirable fish (Allendorf & Hard, 2009; Bowles et al., 2019; Moles et al., 2010), models showing walleye harvests have been above maximum sustainable yields since the early 2000s, and a trend in harvest decline since 2010 (Manitoba Sustainable Development, 2018). The data gathered here, particularly the spatial variation in genes that may drive functional differences among Lake Winnipeg walleye, is useful for generating hypotheses that test and explain organismal responses to environmental stressors, thereby providing additional information for resource managers. For instance, life‐history trait differences can inform conservation in threatened fishes by identifying resilient populations in a system (Hamidan & Britton, 2015). Therefore, possible functional variation identified in this study may underlie heritable genetic differences among Lake Winnipeg walleye that change important traits such as tolerance to environmental conditions and growth rate differences. In addition, with weak population differentiation, the possible functional variation discovered with RNA sequencing may be used in the delineation of conservation units, or population units with adaptive differentiation also considered (Funk et al., 2012). This information may be useful for integrating demographic connectivity and functional differences among walleye into a cohesive management framework.

We have shown how RNA sequencing data can be used for a population genomic scan in a nonmodel fish, even in a system where little molecular information is available. Filtering for Hardy–Weinberg equilibrium and linkage disequilibrium allows investigators to draw neutral markers from mRNA sequence data, making it useful for classical population genetic approaches. By contrast, the wide selective effects present in species’ transcriptomes allow for hypothesis‐generating outlier tests that may reveal variation underlying phenotypic differences among populations. Nonlethal sampling makes RNA sequencing useful for species with low population sizes and for follow‐up studies, such as the potential to link transcriptomic patterns or genetic data with tagged individuals in the wild using telemetry (Jeffries et al., 2014; Miller et al., 2011; Moore et al., 2017). Despite its high cost relative to other genotyping methods, RNA sequencing data can yield physiological in addition to genetic information discussed here. We therefore argue that it is useful for addressing diverse molecular questions in the conservation of freshwater species.

## CONFLICT OF INTEREST

None declared.

## AUTHOR CONTRIBUTION

**Matt J. Thorstensen:** Conceptualization (equal); Data curation (equal); Formal analysis (lead); Methodology (equal); Software (lead); Validation (equal); Visualization (equal); Writing‐original draft (lead); Writing‐review & editing (equal). **Jennifer D. Jeffrey:** Investigation (supporting); Validation (equal); Writing‐review & editing (supporting). **Jason R. Treberg:** Funding acquisition (equal); Investigation (supporting); Methodology (supporting); Project administration (equal); Supervision (equal); Writing‐review & editing (supporting). **Douglas A. Watkinson:** Investigation (equal); Methodology (equal); Resources (equal); Supervision (equal); Writing‐review & editing (supporting). **Eva C. Enders:** Conceptualization (equal); Funding acquisition (lead); Investigation (equal); Project administration (lead); Resources (lead); Supervision (equal); Writing‐review & editing (supporting). **Ken M. Jeffries:** Conceptualization (equal); Data curation (equal); Formal analysis (supporting); Funding acquisition (equal); Investigation (lead); Methodology (equal); Project administration (equal); Resources (equal); Software (equal); Supervision (lead); Validation (supporting); Visualization (equal); Writing‐original draft (supporting); Writing‐review & editing (lead).

### Open Research Badges

This article has been awarded Open Materials, Open Data Badges. All materials and data are publicly accessible via the Open Science Framework at https://www.ncbi.nlm.nih.gov/sra/PRJNA596986; https://github.com/BioMatt/Walleye_RNAseq.

## Supporting information

Supplementary Materials

## Data Availability

Raw sequence reads are available through the National Center for Biotechnology Information Sequence Read Archive (accession #PRJNA596986, https://www.ncbi.nlm.nih.gov/sra/PRJNA596986). Code used for analyses and bioinformatics in this manuscript is available at https://github.com/BioMatt/Walleye_RNAseq.
